# Understanding the Acceptability and Uptake of HPV Self-Sampling Amongst Women Under- or Never-Screened for Cervical Cancer in Toronto (Ontario, Canada): An Intervention Study Protocol

**DOI:** 10.3390/ijerph18179114

**Published:** 2021-08-29

**Authors:** Aisha Lofters, Kimberly Devotta, Vijayshree Prakash, Mandana Vahabi

**Affiliations:** 1 Peter Gilgan Centre for Women’s Cancers, Women’s College Hospital, Toronto, ON M5S 1B2, Canada; Aisha.Lofters@wchospital.ca; 2 MAP Centre for Urban Health Solutions, St. Michael’s Hospital, Toronto, ON M5B 1T8, Canada; 3 Dalla Lana School of Public Health, University of Toronto, Toronto, ON M5T 3M7, Canada; 4 Department of Family and Community Medicine, University of Toronto, Toronto, ON M5G 1V7, Canada; 5 PCCIS Research Project, Ryerson University, Toronto, ON M5B 1Z5, Canada; Vijayshree.Prakash@ryerson.ca; 6 Daphne Cockwell School of Nursing, Ryerson University, Toronto, ON M5B 1Z5, Canada; MVahabi@ryerson.ca

**Keywords:** cervical cancer, cervical screening, HPV self-sampling, community champions, health equity

## Abstract

Cervical cancer remains a global public health concern, even though scientific advancements have made the disease almost entirely preventable. With the link between human papillomavirus (HPV) and cervical cancer, and the subsequent improvement in screening technology, there is potential to improve access and coverage of cervical screening with the introduction of HPV self-sampling. In Ontario, Canada, a province with a cytology-based screening program (i.e., Pap test), women who identify as South Asian, West Asian, Middle Eastern and North African have some of the lowest rates of screening, and research suggests they have a higher burden of cervical cancer. In this study, we will use both quantitative and qualitative methods to understand the acceptability and uptake of a take-home HPV self-sampling kit. Working with community champions—people with pre-existing connections with local groups—we will recruit women from these groups who are under- or never-screened for cervical cancer. Women will self-select whether they are in the group that tries HPV self-sampling or in the group that does not. We will aim for 100 women in each group. All participants will provide feedback on the feasibility, acceptability and preferences for cervical screening through a survey and phone follow-up. Women who self-select the HPV self-sampling group, will be followed up to find out if they followed through with self-sampling and to understand their experience using the device. Women who do not want to try self-sampling will be followed up to see if they went on to get a Pap test. The qualitative phase of this study consists of five focus groups with participants and semi-structured interviews with key informants in the community.

## 1. Introduction

Almost all cases of cervical cancer are caused by Human Papillomavirus (HPV). With appropriate screening (i.e., the Pap test), cervical cancer is highly preventable; accordingly, Canada and other high-income countries with widespread screening have observed significant decreases in incidence and mortality in recent decades [1,2,3,4]. However, screening participation seems to have reached a ceiling over the past two decades in Ontario, Canada’s most populous and diverse province [5].

The purpose of screening is to reduce the risk of cervical cancer by looking for, and treating, lesions that have the potential to become cancerous [6]. Throughout Canada, organised screening programs have used cytology testing and this has been largely responsible for a dramatic decline in cervical cancer incidence, but these rates have now plateaued [7].

The Papanicolaou (Pap) test was introduced in Canada in 1949. By the 1960s, it was being used to opportunistically screen for cervical cancer in Ontario [8]. In 2000, a publicly funded, organised screening program was established in Ontario and during this time, screening rates of eligible women were estimated to be around 59% [8]. Since then, the Ontario Cervical Screening Program (OCSP) has encouraged uptake of screening in the province, seeing its greatest success in 2007–2009 with 67% participation. Since 2013, screening has remained stable around 60%, well below provincial and national targets [8,9]. The current OCSP recommends that everyone with a cervix who has been sexually active commence cytology-based screening at the age of 25 [10].

Increasing international evidence suggests that HPV testing is more accurate and sensitive for detecting pre-cancers, compared to cytology (i.e., a Pap test) [7]. The World Health Organisation (WHO) has set the goal of accelerating the elimination of cervical cancer, using a life-course approach that includes screening with a high-performance test that is equal to or better than an HPV test [11]. In support, the Canadian Partnership Against Cancer has set out a plan to eliminate cervical cancer by 2040 in Canada [12]. Around the world, many jurisdictions have either adopted HPV testing or are considering it for cervical screening. Australia moved to HPV testing in 2016, and other areas are set to follow, including Ontario. In 2013, the OCSP in conjunction with the Program in Evidence Base Care (PEBC)—an initiative of Cancer Care Ontario, the cancer agency arm of Ontario Health—made the recommendation to Ontario’s Ministry of Health that HPV testing represented the best evidence-based strategy for cervical cancer screening and was the most accurate way to screen for cervical cancer precursors [8]. An additional benefit of HPV testing for cervical cancer screening is the option for self-sampling devices.

Previous studies by our research team and others demonstrate that certain subgroups of women in Canada, including immigrants and women of low income, are less likely to be appropriately screened, with South Asian women being at particular risk of under-screening, followed by Middle Eastern and North African women [13,14,15,16,17,18,19,20,21,22,23,24,25,26,27,28,29,30,31,32,33,34,35,36,37,38,39]. These are particularly and persistently true in the province of Ontario where the adjusted odds ratio of screening for South Asian women compared to non-immigrant women was 0.61 (95% CI 0.59–0.64), and 0.68 (95% CI 0.64–0.72) for Middle Eastern and North African women [40].

Low levels of screening among these women have been related to such barriers as lack of a family physician, inconvenient clinic hours, problems with transportation, having a male physician, cultural barriers, (e.g., different cultural norms around modesty, language barriers) and indirect costs associated with screening, (e.g., for childcare, taking time off work) [13,14,15,16,17,18,19,20,21,26,27,28,29,30,32,33,34,35,36,37,38,39]. The persistence of these disparities over decades suggests that innovative methods are needed to address these barriers and improve screening rates for under- or never-screened (UNS) women. HPV self-sampling, when used as a primary screening test to triage women for subsequent Pap testing, is not part of existing screening practices in Canada, but has the potential to be one such innovative method [35,41,42,43,44,45,46]. There is strong evidence of the validity of HPV self-sampling compared to clinician-collected cervical samples, as well as of high acceptance and positive attitudes of women toward self-sampling [47,48,49,50,51,52]. In the Netherlands, the national screening program allows women to request a self-sampling kit if they are uncomfortable with a collection being taken by their provider [53]. In Australia, where they have already implemented HPV testing in their national cervical screening program, self-sampling is currently offered as an option for women who have never been screened or are overdue [54].

Self-sampling as an alternative to a Pap test is being studied amongst populations with markedly low under-screening. Currently, in the United Kingdom, the YouScreen study is offering 31,000 people eligible for cervical screening in north and east London the opportunity to take a self-sample [55]. There are also Canadian studies available on acceptability of self-sampling [35,47,49,53,54,55], with some involving trialing of self-sampling [47,53,55]. For example, self-sampling kits have been tried among under-housed women in British Columbia (BC) [47]. This study aims to determine the feasibility of HPV self-sampling for under- or never-screened (UNS) women of South Asian, West Asian, Middle Eastern and North African ethnicity in Ontario as a method of reducing barriers to cervical cancer screening.

The use of community champions is well-supported in the research literature as an effective means of reaching specific ethnocultural groups to educate and encourage cancer screening, including cervical cancer screening. Therefore, we sought to utilise community champions in this study [56,57,58,59,60,61].

## 2. Research Question

Our specific research questions are: (1) What proportion of UNS women who self-identify as West and South Asian, Middle Eastern or North African and are approached by a community champion will agree to undergo cervical screening, and subsequently use an HPV self-sampling kit? (2) What are the facilitators and barriers to using HPV self -sampling? (3) What are the experiences of women who undergo self-sampling and have a positive result?

## 3. Theoretical Framework

This work will be guided by the RE-AIM (reach, effectiveness, adoption, implementation, maintenance) framework, a comprehensive framework to assess health initiatives in the real-world setting [62].

## 4. Materials and Methods

This study has received Research Ethics Board approvals from St. Michael’s Hospital (REB # 18-058; May 2018) and Ryerson University (REB# 2018-219; June 2018).

### 4.1. Study Participants and Recruitment Strategy

This multi-method study will involve two groups of women: those who do and do not agree to use the HPV self-sampling kit. Eligibility criteria are: self-report of >4 years since last Pap test, including no history of Pap test; women aged 30–69 years, in line with current provincial guidelines on HPV testing for cervical cancer screening [2]; have ever been sexually active; self-identifying as West or South Asian, Middle Eastern or North African; living in the Greater Toronto Area (GTA) in Ontario, Canada; able to communicate in English; able to provide informed consent and willing to share contact information with the study team. In line with the cervical cancer screening guidelines in Ontario, women who have never been sexually active are not eligible to participate. Women who are currently pregnant are also not eligible to participate, as the HPV self-sampling device that will be used in the study has not been trialled with women who are pregnant. Women who have undergone a hysterectomy but retained their cervix are eligible.

Women will be approached at community-based locations (e.g., community centres, faith-based facilities) in Greater Toronto Areas (GTA) with a focus on those in Peel Region, as Peel has a very large South Asian population [63]. We will aim for places in the community that have culturally specific services, programming and events. This will include more formal settings like community health centres, libraries and places of worship, as well as more informal groups such as tea parties, parent associations and neighbourhood social circles. Recruitment will be led by community champions. In this study, a community champion is someone who is a South or West Asian, North African or Middle Eastern woman herself, who has pre-existing connections with local community groups in Peel and other parts of the GTA. Women will be informed about the study at these locations that are familiar to them through presentations and posted flyers. Women can either communicate their intention to participate directly to a community champion or call in to the study phone line. Those women who are interested in participating or in learning more will meet with the team member in a one-on-one setting of their choosing to discuss the study.

At the time of recruitment, participants will be screened by either the community champions or the study research coordinator using a set of questions to determine their eligibility to be in the study. All participants can choose whether they wish to be interviewed by a community champion or another member of the research team. This option is there in case participants are uncomfortable being interviewed by someone they may know. Since we also recognise that women are more likely to prefer to speak about such a sensitive topic with someone they know, they may choose who consents and interviews them.

All participants will provide either written consent if they are participating face-to-face or verbal consent if they are participating over the phone.

### 4.2. Study Procedures

Women will be divided into two self-selected cohorts. Cohort A will consist of eligible women who are willing to use the HPV self-sampling kit. Cohort B will consist of eligible women who do not agree to use the kit but consent to participate in the study. We anticipate it will be feasible to recruit a maximum of 100 women in each cohort over the two-year study period. Women in both cohorts will complete an interviewer-administered questionnaire that queries demographics, medical history, attitudes and cervical cancer screening practices. At the time of the questionnaire, all women will receive cervical cancer screening educational material from Ontario Health (Cancer Care Ontario)—the provincial cancer agency that oversees Ontario’s cancer screening programs.

### 4.3. Self-Sampling Process for Cohort A

Women in Cohort A will be provided with a HerSwab HPV self-sampling kit and asked to use it at a convenient time following verbal instructions on its use and on the mailing procedure. The HerSwab™ is a class 2 medical device approved by Health Canada (MDL license 94847). Eve Medical, the manufacturer of HerSwab, is an accredited ISO13485 medical device manufacturer. Women will receive this kit either through the community champion during in-person recruitment or in the mail when recruited over the phone.

Women will be informed that the kits should be received by the laboratory as soon as possible, but particularly within two weeks of the sample being taken in order to be accurately processed. A follow-up phone call approximately one week after being given the kit will serve to both remind women to mail the kit and to query if they have used it about their views on ease of use, clarity of instructions and willingness to use for future screening. Participants will be telephoned a maximum of three times in order to speak to them directly. If another person answers the telephone, he/she will be told that [research team member name] from St. Michael’s Hospital is calling for the participant about a study, and a return phone number will be left. No other information will be provided in the message. Women who have not yet returned the kit will be called weekly over 3 weeks to remind them to send their kits. After this point, they will not be contacted further with reminders.

Women who we are unable to successfully contact after multiple attempts (e.g., phone number is out of service, household member not passing along message, voicemail not set up, voicemail not personalised) will be mailed a follow up letter that asks them to get in contact with the team.

All kits will be processed at the Mount Sinai Hospital microbiology laboratory in Toronto, Ontario, and women will be provided with a stamped envelope to return the kit to the laboratory. Test results will be electronically faxed directly to the research team, who will then inform the woman of her results, as well as her primary care provider if contact information for one is provided. Test results take 2–4 days on average. Women with negative results will receive a letter from the research team informing them to have a routine screening in five years, in line with provincial recommendations on HPV testing. Their provider will also receive a letter from the research team if the woman provides his/her contact information. Women with positive results will receive a letter from the research team advising them that they need to follow up with their provider for a Pap test, and will also receive a phone call from a research team member. Women with a positive result who have no primary care provider or do not wish to see their regular provider will be assisted with arranging a visit at a walk-in clinic or community health centre in their local area and instructed to bring their results letter. Women will also be connected to the HealthCareConnect program to help them find a long-term primary care provider if they are interested. All women with positive results will be contacted 3 months later to determine what their follow-up was, including whether they connected with a provider and whether they got a Pap test. All women in Cohort A will be compensated CAD 30 for their participation.

### 4.4. Process for Cohort B

Women in Cohort B will answer questions on their reasons for not wanting to try the kit. They will also be contacted 3 months later for a brief telephone survey to determine whether they went on to have a Pap test after reviewing the educational pamphlets dispensed at the beginning of the study. Similarly to Cohort A, women who we are unable to successfully contact will receive a letter in the mail that asks them to get into contact with the team. All women in Cohort B will also be compensated CAD 30 for their participation. In the event that a participant in Cohort B changes her mind and wishes to try the kit she will be moved to Cohort A and provided with a kit and asked the additional questions on reasons for wanting to try the HPV self-sampling kit.

**Figure 1:**
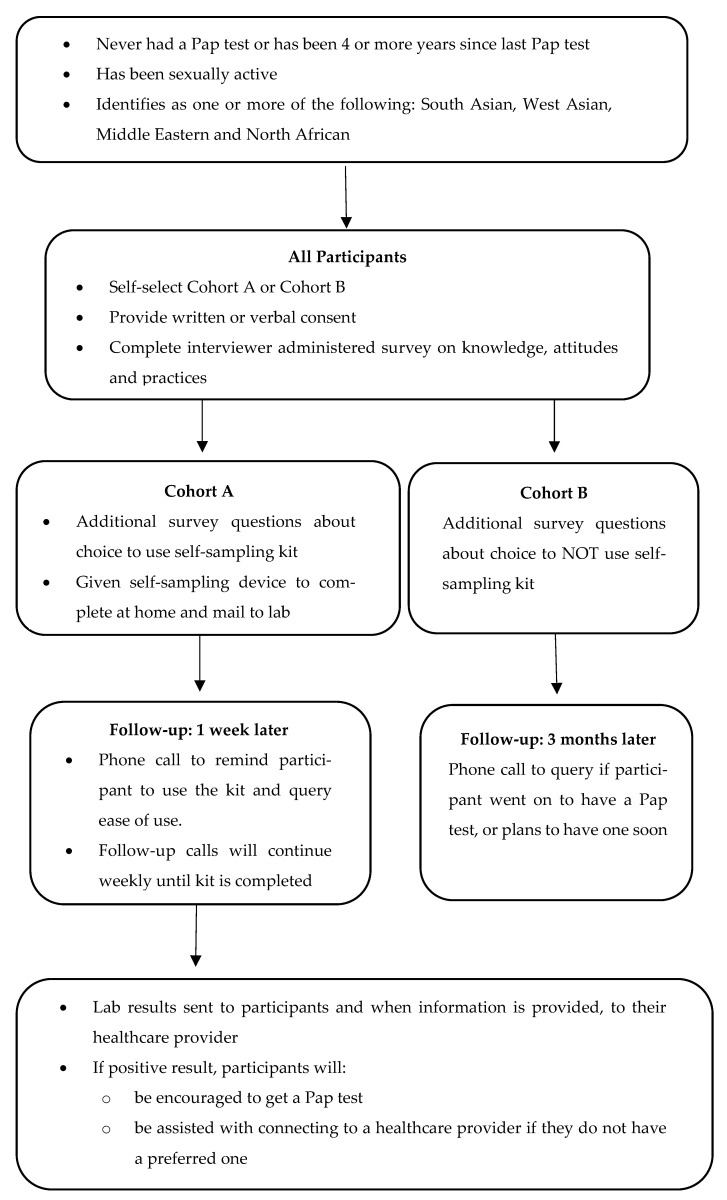
Recruitment and participation for Cohort A and Cohort B.

The process from recruitment to screening and then to study completion, is summarised in Figure 1.

### 4.5. Qualitative Work

We will hold five focus groups in this study to understand adoption, implementation and maintenance. Three focus groups will be held with women from Cohort A to explore their experiences, barriers and facilitators in using HPV self-sampling and suggestions to improve access and uptake. We will randomly select women from cohort A to invite until we are able to recruit 8–10 women per focus group. Depending on the number, women in Cohort A who receive a positive test result will either be interviewed or grouped together, to focus specifically on follow-up after a positive result.

Two focus groups will be held with women in Cohort B to explore barriers and facilitators to use of the kit and attitudes toward cervical screening. Women will be recruited by the same method as for Cohort A.

Women will be invited by phone and/or email to be a part of the focus group. All focus groups will be facilitated by at least two members of the research team, including the community champion who is fluent in multiple languages in case interpretation is required at times. Women in focus groups will be compensated CAD 30 for participation. One-on-one interviews will be arranged for women who have concerns about participating in focus groups. One-on-one interviews will also be scheduled for women who are unable to attend a focus group and/or the next focus group will be a while away from when they participated in the questionnaire (e.g., if a woman participated in the winter and the next focus group is in the summer, we will want to interview her before the summer).

We will also be conducting key informant interviews with people in the community that are involved or consulted with during recruitment to inform research question 2 and the assessment of adoption and maintenance. This includes the community champion and leaders/managers of groups where we recruited our participants. All interviews will take place over the phone. All interviewees will complete verbal consent after reading the letter of information. The interviews will be semi-structured, using a pre-determined schedule of questions to guide the conversations.

### 4.6. Data Collection

To answer research question 1 and assess reach and effectiveness, we will track outreach statistics by keeping counts of the number of women approached, the number of women that consented, the proportion of study participants who self-select into each cohort, the proportion of women in Cohort A who: (i) mail in the kit, (ii) report willingness to use the kit again and (iii) test HPV positive, and the proportion of women in Cohort B who self-report going for a screening Pap test.

The survey will first ask participants a series of demographic questions. Participants will then be asked a series of ‘true or false’ questions with the objective of understanding their knowledge of cervical cancer. A series of statements along with a 5-point Likert scale ranging from ‘strongly disagree’ to ‘agree’ will be used to understand participants’ attitudes around cervical cancer. Lastly, participants will be queried about their history of being screened for cervical cancer, (i.e., getting a Pap test), as well as their reasoning behind their decision to use or not use a self-sampling device, and thoughts on what others may like or not like about the device. All surveys will be collected on electronic tablets, using web-based forms programmed in Qualtrics.

Qualitative data collected during the focus group will include barriers and challenges in using HPV self-sampling, motivators that should be offered in conjunction with screening, identification of self-sampling predictors and major barriers in undertaking self-sampling among those who refused (research question 2). It will also understand the experience and follow-up actions of women who try self-sampling and receive positive test results (research question 3).

### 4.7. Privacy and Confidentiality

Electronic study data collected in the field will be collected on encrypted laptops or tablets with cellular service, (i.e., not using WiFi) and downloaded to St. Michael’s Hospital network. Data will be retained for 7 years after the study has finished in line with hospital research ethics board policies.

During focus groups and interviews, participants will be encouraged not to give their name on the audio-tape and an alpha numeric study number will be assigned when their audiotape is transcribed. Since it is possible that participants may disclose identifying information, all such identifying information will be changed in the written transcripts of the interviews and in any written reports or oral presentations so that participants’ privacy will be protected. Any quotations used in written reports or publications will be showcased such that individual participants cannot be identified from the information contained within the quotation. All audio recordings will be transcribed by a transcription service that has signed a confidentiality agreement with the hospital. Only the study team will have access to the study data. All data will be stored according to institutional research data storage policies.

## 5. Data Analysis

Univariate descriptive statistics will be used to describe the study participants based on survey responses. Bivariate and multivariate analyses, where sample size allows, will be conducted to determine variables associated with HPV self-sampling uptake and predictors of screening practices. Audio-taped focus groups and interviews will be transcribed and analysed by inductive thematic analysis using a reciprocal coding approach [64]. In doing so, each transcript is first reviewed independently, and then through dialogue, composite themes are developed by at least two researchers. Thematic analysis is a method for grouping diverse sections of data into smaller analytic units [65]. A coding framework will be developed. Descriptive summary using both themes (common patterns) and illustrations (unique aspect of experience) will be reported. Triangulation of methods will be used for systematic comparison and verification of study findings, considering the potential for complementarity, initiation and expansion

## 6. Strengths and Limitations

A main strength of our study design is our engagement of community champions in the recruitment and data collection process. For women who are South or West Asian, Middle Eastern and North African that are under- or never-screened, the topic of cervical cancer may be uncomfortable or seldom discussed. Language skills and sensitive approaches to topics of sexual health, reproductive organs and cancer factor into screening decisions [15,66,67,68,69]. Stigma within some of these ethnic communities around sexual activity, virginity and marital status can also pose barriers, as a woman getting screened may then imply certain details about their sexual activity [17,66,70,71]. Peers can play an impactful role in the uptake of screening when involved in education and facilitation of appointments [60]. In addition to their meaningful connections with women in the study, their knowledge of spaces and gatherings where people feel comfortable to discuss personal matters, means that we can move conversations and recruitment away from more traditional spaces that may not currently be effective for this group of women, (e.g., doctor’s offices), to spaces where women may be more comfortable to talk to friends and other peers. It is our aim that this study design takes a new approach to awareness and facilitation of screening, to harness the expertise of peers in the community and to meet under- or never-screened women in spaces where they may feel more comfortable to discuss seemingly sensitive or personal topics.

While our community champions hold skills for many different languages spoken in South and West Asian, Middle Eastern and North African countries, there are still some languages that we may not be able to accommodate and would therefore need an interpreter. We recognise that the introduction of an interpreter may impact recruitment and comfort levels. Amongst our target group of women, there is much diversity along such lines as ethnicity, religion, age, social class, sexual orientation, education and marital status. When we consider a ‘peer’, it is important to recognise that participants will relate to the community champions differently. Some may experience more relatability and comfort than others, and this may impact who is recruited. We do, however, believe we will be able to reach more people than we would without the engagement of community champions. We also believe that the community champions already hold a certain level of recognisability within the spaces where they are recruiting, and this will be quite effective for engagement.

## 7. Conclusions

The results from this community-based study will provide valuable insights for the implementation of HPV self-sampling, and the improvement of uptake of cervical cancer screening more broadly, for UNS women from South Asian, West Asian, Middle Eastern and North African countries, a substantial proportion of residents of Ontario.
